# Transcranial Direct Current Stimulation Does Not Enhance Perceptual Learning of Chinese Character Reading in Adults With Macular Degeneration

**DOI:** 10.1167/iovs.67.1.16

**Published:** 2026-01-08

**Authors:** Anqi Lyu, Melanie A. Mungalsingh, Andrew E. Silva, Shamrozé Khan, Tammy Labreche, Susan J. Leat, George C. Woo, Stanley Woo, Benjamin Thompson, Allen M. Y. Cheong

**Affiliations:** 1 School of Optometry, The Hong Kong Polytechnic University, Hong Kong, China; 2 School of Optometry and Vision Science, University of Waterloo, Waterloo, Ontario, Canada; 3 Centre for Eye and Vision Research (CEVR), Hong Kong, China; 4 Research Centre for SHARP Vision, The Hong Kong Polytechnic University, Hong Kong, China

**Keywords:** AMD, tDCS, non-invasive brain stimulation, perceptual learning, reading

## Abstract

**Purpose:**

Macular degeneration impairs central vision, compelling patients to use their peripheral vision for reading, which is difficult due to reduced spatial resolution and crowding. Although perceptual learning improves reading, single-session anodal transcranial direct current stimulation (a-tDCS) over the visual cortex has shown inconsistent outcomes, with transient improvements observed in English reading but no benefit for Chinese reading in macular degeneration patients. This randomized controlled trial investigated whether combining multi-session a-tDCS with perceptual learning enhances Chinese reading performance in these patients compared to sham stimulation.

**Methods:**

Twenty Chinese-reading patients with macular degeneration (39–90 years old) were randomized to receive either active (*n* = 10) or sham (*n* = 10) a-tDCS during six sessions of rapid serial visual presentation (RSVP) reading training. Trained outcomes (RSVP reading) and untrained functions (sentence reading, crowding, contrast sensitivity, and visual acuity) were compared at baseline, 1 day, and 1 month post-training.

**Results:**

Perceptual learning significantly improved RSVP reading speed (*P* < 0.001) in both groups, with effects lasting at least a month. No additive effect of active versus sham a-tDCS was observed (group × time *P* = 0.99). Transfer effects to untrained functions were limited to visual acuity and critical print size for sentence reading.

**Conclusions:**

Perceptual learning enhances Chinese reading performance in individuals with macular degeneration, but a-tDCS confers no additional benefit. This contrasts with previous results where non-invasive brain stimulation enhanced English reading in macular degeneration. The results emphasize the need for more refined neuromodulation strategies for improving logographic reading.

Macular degeneration is a leading cause of irreversible central vision loss, significantly affecting patients’ daily activities, including reading,^1^^,^^2^ which requires precise visual resolution^3^ and oculomotor control.^4^ Patients with central scotomas must rely on a paracentral or peripheral retinal locus for vision. However, peripheral vision is inherently limited by reduced spatial resolution,^5^^,^^6^ poorer eye movement control,^7^^,^^8^ restricted visual span,^9^^–^^11^ and increased crowding effects,^12^^,^^13^ all of which hinder reading performance. Although interventions such as perceptual learning aim to improve eccentric viewing efficiency and show promise, their effects are often slow (see Park et al.^14^ for a review). In contrast, neuromodulation techniques, such as transcranial direct current stimulation (tDCS), offer a promising alternative to enhance neuroplasticity and hasten functional recovery.^15^^–^^17^ Despite these plausible rehabilitative benefits, the optimal integration of tDCS with perceptual learning remains underexplored, especially in patients with macular degeneration, where cortical reorganization^18^ and residual visual plasticity^19^^,^^20^ may offer untapped potential for rehabilitation.

The tDCS technique involves the application of a mild electrical current of 1 to 2 mA through head-mounted electrodes, inducing regional changes in cortical excitability and neurotransmitter concentration that outlast the stimulation.^21^ Anodal tDCS (a-tDCS) applied to the visual cortex has generated transient improvements in English reading speed following a single 20-minute session in patients with macular degeneration.^22^ However, it did not enhance Chinese reading performance in either macular degeneration patients^22^ or individuals with normal peripheral vision.^23^ This discrepancy may stem from the distinct visual-processing demands of logographic versus alphabetic scripts. Chinese reading relies more on global configuration processing to resolve fine character details,^24^^,^^25^ a task typically mediated by foveal vision but compromised in peripheral vision.^11^ This limitation suggests that a single session of a-tDCS, which temporarily modulates plasticity, may be insufficient for enhancing logographic processing, highlighting the possible importance of cumulative dosing for sustained functional gains.

Perceptual learning, which involves repetitive, task-specific training to achieve sensory function changes, has induced consistent improvements in studies of reading rehabilitation for both Chinese^26^ and English.^10^^,^^19^^,^^27^^–^^29^ By having patients repeatedly read sentences or letters, perceptual learning increases reading speed in individuals with macular degeneration.^19^^,^^27^^,^^28^ However, these effects require repeated training sessions over extended periods—often spanning weeks or months—and are highly specific to the trained task and stimuli.^30^^–^^32^ As a result, the general efficacy of perceptual learning for enhancing peripheral reading has remained limited.^19^^,^^27^^,^^33^ Because a-tDCS may lower the threshold for neuroplastic changes,^34^ integrating it with perceptual learning paradigms may help address the challenges associated with perceptual learning alone. Studies have reported that a-tDCS enhances perceptual learning of texture discrimination^35^ and motion perception^36^ in normally sighted individuals and stereopsis in adults with amblyopia,^37^ compared to sham-controlled training paradigms. However, no studies have yet investigated whether repeated a-tDCS sessions combined with perceptual learning could benefit reading rehabilitation in patients with macular degeneration, despite their reliance on peripheral vision and the critical need for effective interventions.

This study aimed to address the knowledge gap by testing the hypothesis that active a-tDCS would enhance perceptual learning of a Chinese reading task in macular degeneration patients compared to perceptual learning combined with sham stimulation. This study also explored whether the training effects would transfer to a range of untrained visual functions in the active a-tDCS group. By focusing on Chinese reading, a task where a single session of a-tDCS has shown no effect,^22^ this study aimed to advance the understanding of how repeated neuromodulation protocols could be utilized for complex logographic reading. In addition, it sought to inform future rehabilitation approaches for macular degeneration patients, bridging the gap specifically between neuromodulation research and clinical application.

## Methods

### Participants

Participants were recruited from local hospitals and research centers in Hong Kong in accordance with a pre-registered protocol (ClinicalTrials.gov ID: NCT04762368). The trial initially aimed to include both Chinese- and English-reading populations, but the analysis herein focused exclusively on Chinese-reading participants due to regional recruitment challenges. Twenty participants with bilateral macular degeneration, 18 with age-related macular degeneration (AMD) and two with juvenile macular degeneration (JMD), completed the study (see Supplementary Material for the CONSORT flow diagram). All participants were native Cantonese speakers and fluent readers of traditional Chinese. Eligibility criteria included (1) absence of significant ocular diseases affecting visual function, aside from macular degeneration; (2) no history of neurological or psychiatric illness; and (3) no contraindications to a-tDCS such as epilepsy or cranial implants. Participants were randomized into either the experimental group (active a-tDCS + perceptual learning, *n* = 10) or the control group (sham a-tDCS + perceptual learning, *n* = 10), with stratification based on macular degeneration type (AMD or JMD) and visual acuity (<0.6 or ≥0.6 logMAR). Baseline demographics and clinical characteristics did not differ significantly between groups (Table). All participants gave written informed consent after receiving an explanation of the nature and possible consequences of the study. The study was approved by the Departmental Research Committee of the School of Optometry at the Hong Kong Polytechnic University (HSEARS20190716005) and adhered to the tenets of the Declaration of Helsinki.

**Table.** Demographic Data of the Participants

	Experimental Group (*n* = 10)	Control Group (*n* = 10)	Differences Between Groups
Age (y), mean ± SE (range)	67.29 ± 4.18 (39–82)	69.07 ± 3.97 (40–90)	*t* = 0.31; *P* = 0.76
Sex (female/male), %	30/70	10/90	*U* = 40; *P* = 0.58
Macular degeneration type, %			*U* = 50; *P* = 1.00
AMD	90	90	
JMD	10	10	
Disease duration (y), mean ± SE (range)	7.00 ± 1.99 (1–20)	8.90 ± 1.33 (2–17)	*t* = 0.81; *P* = 0.44
Best-corrected visual acuity (logMAR), mean ± SE			
Distance	0.52 ± 0.10	0.59 ± 0.10	*t* = 0.46; *P* = 0.65
Near	0.56 ± 0.10	0.61 ± 0.11	*t* = 0.35; *P* = 0.73
Contrast sensitivity (log CS), mean ± SE	1.01 ± 0.08	0.78 ± 0.08	*t* = 2.04; *P* = 0.06
Character reading, mean ± SE			
Maximum reading speed (log characters/min)	2.38 ± 0.08	2.22 ± 0.08	*t* = 1.33; *P* = 0.20
Critical print size (logMAR)	1.23 ± 0.12	1.44 ± 0.13	*t* = 1.24; *P* = 0.23
Sentence reading, mean ± SE			
Maximum reading speed (log characters/min)	2.04 ± 0.14	1.84 ± 0.18	*t* = 0.88; *P* = 0.40
Critical print size (logMAR)	1.05 ± 0.10	1.24 ± 0.08	*t* = 1.42; *P* = 0.17
Reading acuity (logMAR)	0.85 ± 0.12	0.98 ± 0.11	*t* = 0.81; *P* = 0.43

AMD, age-related macular degeneration; JMD, juvenile macular degeneration; Log MAR, logarithm of the Minimum Angle of Resolution measured using an Early Treatment Diabetic Retinopathy Study (ETDRS) chart at 4m (distance) and 40cm (near).

Two-tailed Student’*s t*-test or Mann–Whitney *U* test was used to compare differences between groups.

### Experimental Design

The study employed a between-subjects, double-masked, randomized, placebo-controlled design. Participants attended nine visits over 7 to 10 weeks, including one pre-training assessment, six training sessions, and two post-training assessments at 1 day and 1 month post-training. All assessments and training sessions were performed monocularly using the participant's better-seeing eye (see Supplementary Material for selection procedures) with optimal refractive correction. This approach aligns with previous studies investigating perceptual learning in low-vision participants to ensure that the observed improvements could be attributed to neuroplastic changes within the trained eye rather than to adaptations in binocular coordination or a shift in ocular dominance.^38^^,^^39^ The non-tested eye was occluded with an eye patch.

### Interventions

#### Anodal tDCS Protocol

Active or sham a-tDCS was administered using a battery-powered current stimulator (nurostym tES; Neuro Device Group, Warsaw, Poland), with two 5 × 5-cm rubber electrodes placed inside saline-soaked sponges. The anode was positioned over the occipital poles (Oz, 10–20 electroencephalogram system), and the cathode was placed on a randomly selected cheek.^22^^,^^23^ Both electrodes were secured using an adjustable cap. Active a-tDCS consisted of 2-mA current delivered over 25 minutes, with 30-second ramp-up and ramp-down periods. Sham a-tDCS included only the ramp periods to simulate the tingling sensation, without providing continuous current.

#### Perceptual Learning

The rapid serial visual presentation (RSVP)^3^^,^^12^^,^^19^^,^^40^ method was selected as the primary training and outcome measure because it isolates the perceptual components of reading (e.g., character recognition and processing speed) by presenting characters sequentially at a fixed location. This approach eliminates the confound of unstable eye movements in individuals with macular degeneration.^41^^,^^42^ Chinese sentences (15 characters each, mean stroke count = 8.69 ± 1.11; see Supplementary Material for the sentence selection process) were displayed centrally on a 24-inch monitor (BenQ XL2540, 120-Hz refresh rate, 1920 × 1080-pixel resolution; Zowie, Taipei, Taiwan) at a 65-cm viewing distance, one character at a time. Participants were instructed to look at the center of the screen using their habitual preferred retinal locus (PRL) to view the stimuli. Each training session included two blocks: one combined with a-tDCS (active/sham) and another without stimulation. For each block, four print sizes and four presentation speeds were tested across four interleaved trials. The training print sizes and durations were chosen adaptively relative to each participant's critical print size (CPS), including two smaller sizes, one equal size, and one larger size, to ensure that the task remained challenging yet achievable (see Supplementary Material for the details of the adaptive staircase and curve fitting). Each session lasted around 60 minutes. A total of six sessions^19^ were conducted over 3 weeks, with two sessions per week, except for one participant in the sham a-tDCS group who attended the training weekly and completed the sessions over 6 weeks.

### Pre- and Post-Training Outcome Measures

Participants’ character reading performance was assessed using the RSVP method,^3^^,^^12^^,^^19^^,^^40^ with five print sizes tested at five presentation speeds across at least four trials. The primary outcome was the maximum reading speed (MRS) measured in log characters per minute (log cpm). CPS was defined as the smallest print size at which participants achieved their MRS. Additional secondary outcomes included the following:

1. Sentence reading—Participants read 12-character sentences using Chinese MNRead acuity charts.^43^ Reading speed and errors (omissions/substitutions) were recorded to calculate MNRead log MRS, CPS, and reading acuity, calculated as the smallest print size attempted + (total character errors × 0.008).^44^
2. Crowding effect—Using the Freiburg Vision Test (FrACT),^22^^,^^45^ participants identified the orientation of Landolt C optotypes under isolated (uncrowded) and ring-surrounded (crowded) conditions, both at 3 meters. The crowding effect was calculated as the difference in logMAR thresholds between the two conditions.
3. Contrast sensitivity—The FrACT was used to measure log contrast sensitivity (log CS) via Landolt C identification at varying contrasts.
4. Visual acuity—Best-corrected visual acuity was measured using high-contrast Early Treatment Diabetic Retinopathy Study (ETDRS) charts at 4 meters.

### Statistical Analysis

Statistical analysis was performed in R 4.4.2 using the rstatix and ARTool packages (R Foundation for Statistical Computing, Vienna, Austria). Between-group differences in baseline characteristics were examined using independent Student's *t*-tests for normally distributed variables and Mann–Whitney *U* tests for sex and macular degeneration type (see the Table). For primary and secondary outcomes, time (pre-training vs. 1 day post-training vs. 1 month post-training) × group (active vs. sham) interactions were evaluated using repeated-measures analysis of variance (ANOVA) for normally distributed data or Aligned Rank Transform (ART) for nonparametric ANOVA^46^ when assumptions of data sphericity or normality were violated. *P* < 0.05 was considered statistically significant in a two-tailed test. Any significance was examined using Bonferroni multiple comparison post hoc tests.

## Results

### Effects of a-tDCS Combined With Perceptual Learning on the Primary Outcome

**Figure 1.**
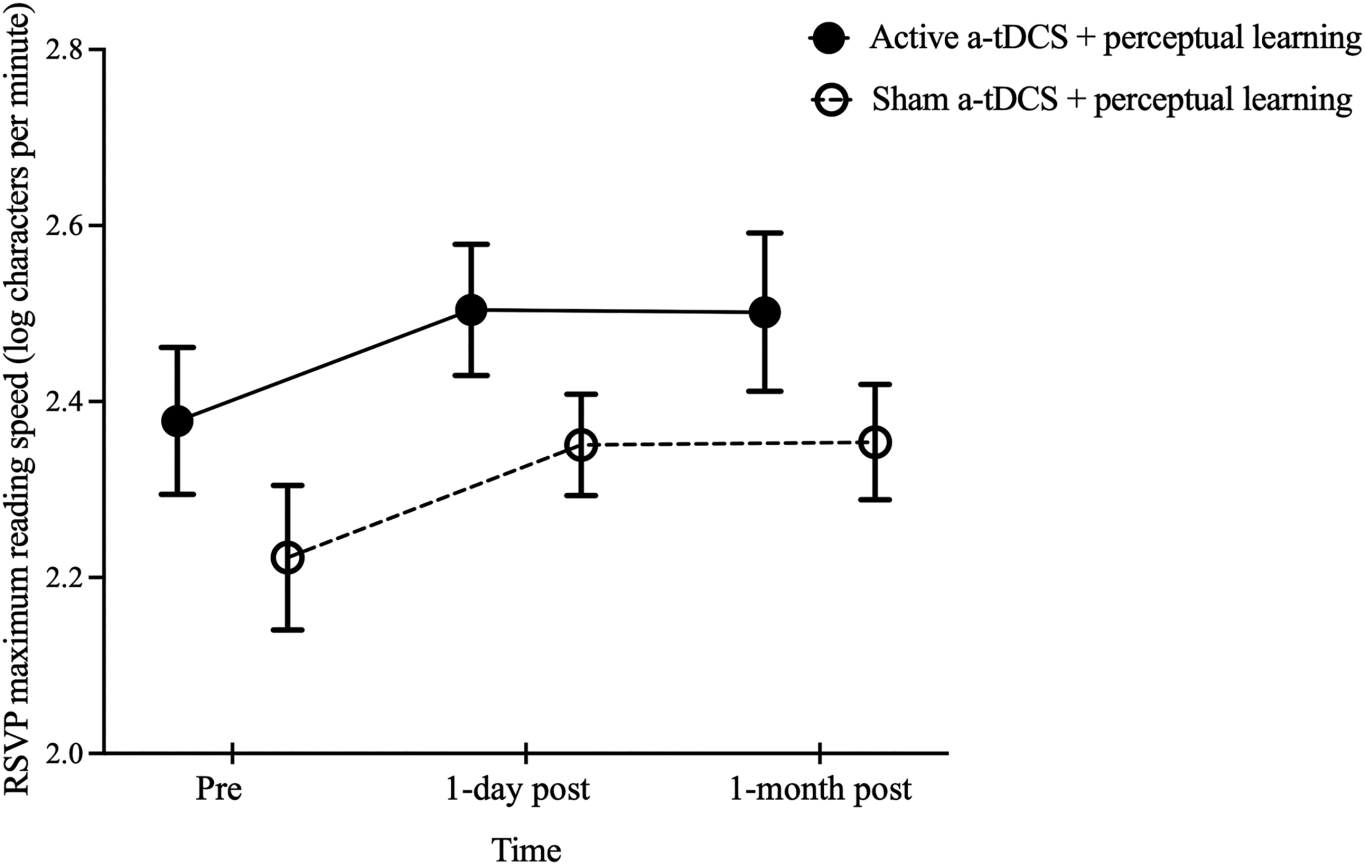
Comparison of RSVP MRS before and after training in the active and sham a-tDCS groups. The RSVP MRS values before training (pre) and at 1 day and 1 month post-training are shown. The *black solid* and *open circles* represent the mean MRS values for the groups that received active and sham a-tDCS, respectively. *Error bars*: ±1 SEM.

Mean character reading speed was assessed at baseline and at 1 day and 1 month post-training. A significant effect of time was found, *F*(2, 36) = 12.02, *P* < 0.001. However, neither the main effect of group, *F*(1, 18) = 2.22, *P* = 0.15, nor the group × time interaction, *F*(2, 36) = 0.01, *P* = 0.99, reached significance. Post hoc comparisons indicated significant improvements in MRS for both groups at 1 day post-training (active a-tDCS, 2.50 ± 0.07 log cpm; sham a-tDCS, 2.35 ± 0.06 log cpm; *P* < 0.01) and at 1 month post-training (active a-tDCS, 2.50 ± 0.09 log cpm; sham a-tDCS, 2.35 ± 0.07 log cpm; *P* < 0.01) compared to baseline (active a-tDCS, 2.38 ± 0.08 log cpm; sham a-tDCS, 2.22 ± 0.08 log cpm). No differences were observed between the 1-day and 1 month post-training tests (*P* > 0.99) (Fig. 1).

### Effects of a-tDCS Combined With Perceptual Learning on Secondary Outcomes

**Figure 2.**
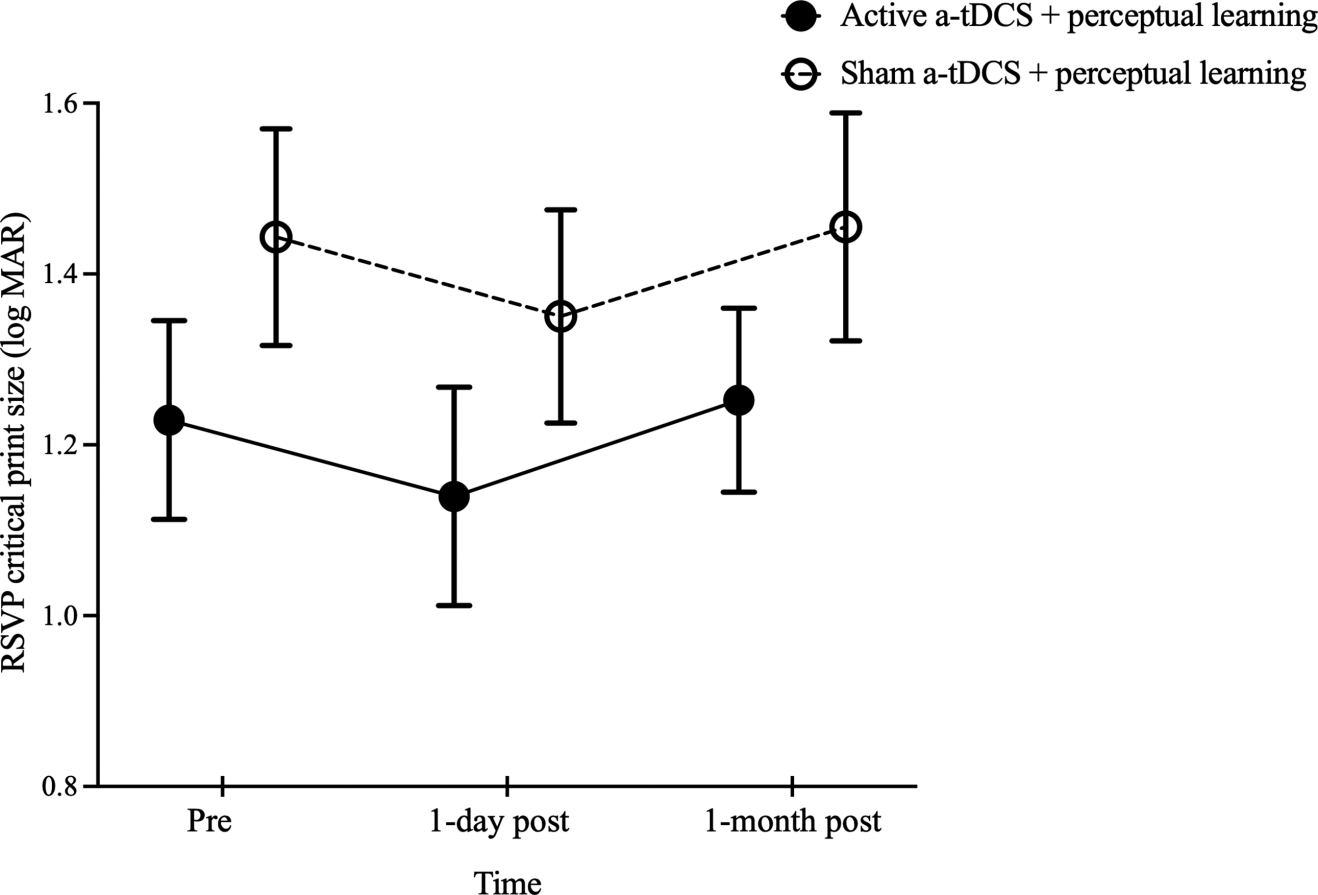
Comparison of RSVP CPS before and after training in the active and sham a-tDCS groups. The RSVP CPS values before training (pre) and at 1 day and 1 month post-training are shown. The *black solid* and *open circles* represent the mean CPS values for the groups that received active and sham a-tDCS, respectively. *Error bars*: ±1 SEM.

The results for RSVP CPS showed that time had a significant effect, *F*(2, 36) = 4.91, *P* = 0.01, whereas neither the group, *F*(1, 18) = 1.54, P = 0.23, nor the interaction effect, *F*(2, 36) = 0.02, *P* = 0.98, reached significance. Post hoc comparisons showed a trend toward a smaller CPS at 1 day post-training for both groups (active a-tDCS, from 1.23 ± 0.12 to 1.14 ± 0.13 logMAR; sham a-tDCS, from 1.44 ± 0.13 to 1.35 ± 0.12 logMAR; *P* = 0.06). However, by 1 month post-training, CPS increased significantly compared to the 1-day post-measurement (1.25 ± 0.11 and 1.46 ± 0.13 logMAR for active and sham a-tDCS groups, respectively; *P* = 0.02). No difference was observed between pre-training and 1-month post-training assessments (*P* > 0.99) (Fig. 2).

**Figure 3.**
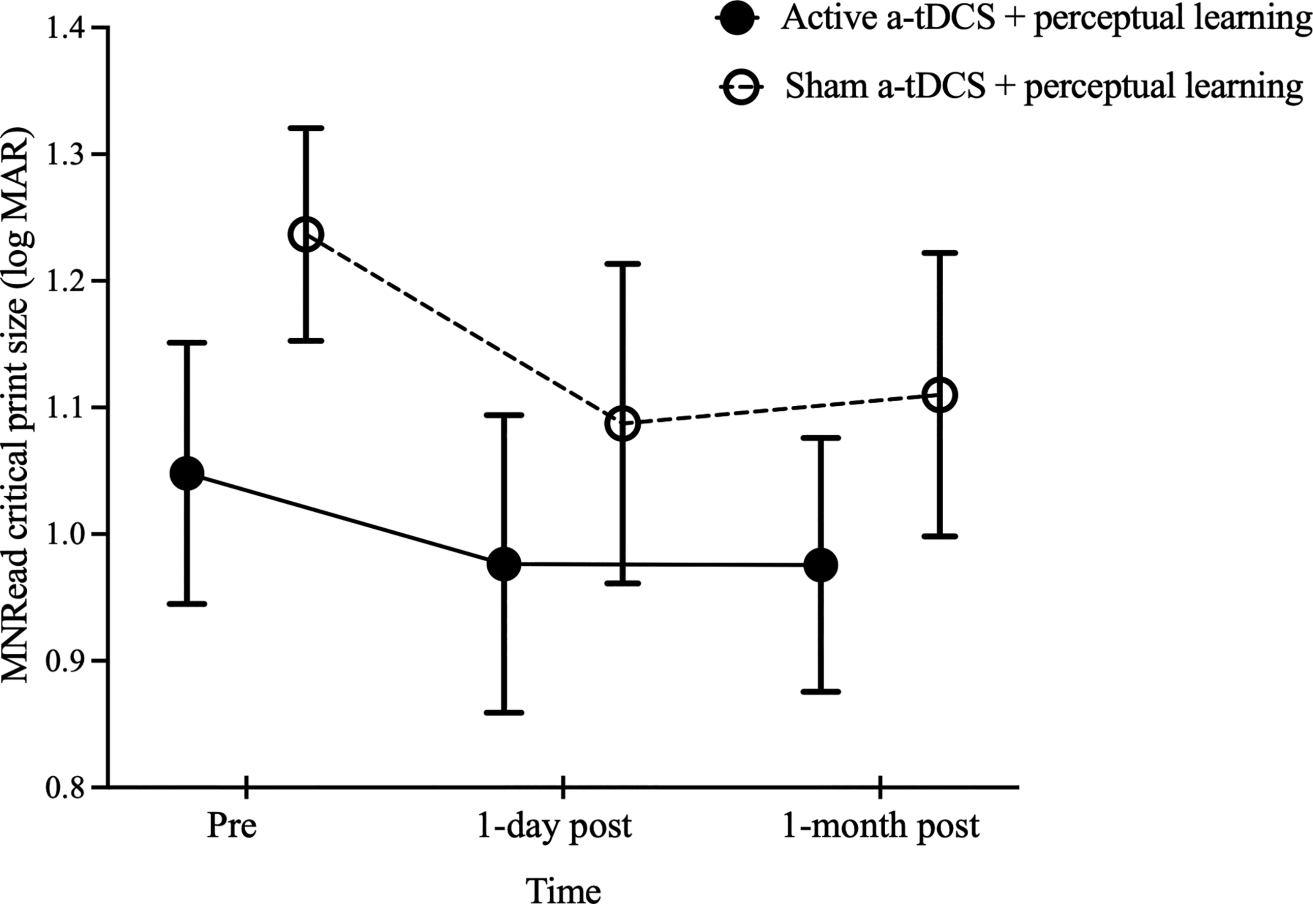
Comparison of MNRead CPS before and after training in the active and sham a-tDCS groups. The MNRead CPS values before training (pre) and at 1 day and 1 month post-training are shown. The *black solid* and *open circles* represent the mean CPS values for the groups that received active and sham a-tDCS, respectively. *Error bars*: ±1 SEM.

For sentence reading performance assessed using Chinese MNRead acuity charts, Shapiro–Wilk tests indicated non-normal distributions for most log MRS data across both active and sham a-tDCS groups (*P* < 0.05), leading to the use of ART ANOVA. The analysis revealed no significant effects of group, ART *F*(1, 18) = 3.21, *P* = 0.09; time, ART *F*(2, 36) = 0.11, *P* = 0.90; or group × time interaction, ART *F*(2, 36) = 1.41, *P* = 0.26, on log MRS (active a-tDCS, 2.04 ± 0.14 log cpm vs. 2.08 ± 0.14 log cpm vs. 2.13 ± 0.10 log cpm; sham a-tDCS, 1.84 ± 0.18 log cpm vs. 1.91 ± 0.07 log cpm vs. 1.84 ± 0.10 log cpm at pre-training and at 1-day and 1-month post-training assessments, respectively). Similarly, there were no significant effects of group, *F*(1, 18) = 1.08, *P* = 0.31; time, *F*(2, 36) = 1.24, *P* = 0.30; or time × group interaction, *F*(2, 36) = 1.75, *P* = 0.19, on MNRead reading acuity (0.92 ± 0.08 logMAR vs. 0.89 ± 0.09 logMAR vs. 0.90 ± 0.07 logMAR at pre-training and at 1-day and 1-month post-training assessments, respectively). In contrast, MNRead CPS demonstrated a significant effect of time, *F*(2, 36) = 4.91, *P* = 0.01, but no group effect, *F*(1,18) = 0.98, *P* = 0.34, or interaction effect, *F*(2, 36) = 0.53, *P* = 0.59. Both groups had smaller CPS at 1 day (1.03 ± 0.08 logMAR; *P* = 0.02) and 1 month post-training (1.04 ± 0.07 logMAR; *P* = 0.04), compared with pre-training (1.14 ± 0.07 logMAR) (Fig. 3).

**Figure 4.**
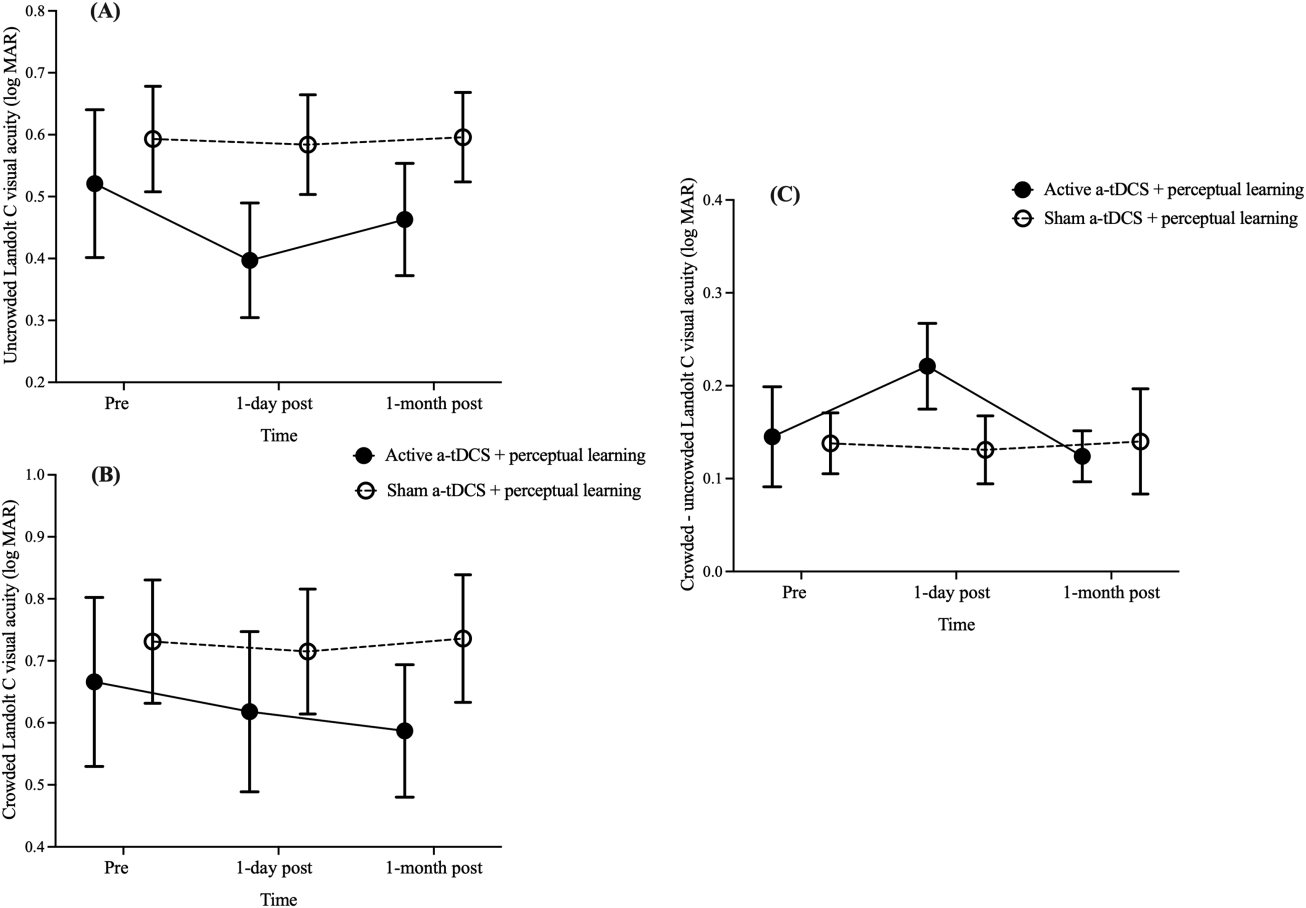
Pre-training versus post-training comparison of uncrowded acuity, crowded acuity, and crowding effect with the FrACT. (**A**) Uncrowded visual acuity. Shown are Landolt C recognition thresholds (logMAR) before and after training for active (*black solid circles*) and sham (*open circles*) a-tDCS groups. (**B**) Crowded visual acuity. Shown are Landolt C recognition thresholds (logMAR) before and after training for active (*black solid circles*) and sham (*open circles*) a-tDCS groups under crowded conditions. (**C**) Crowding effect. Shown are differences between crowded and uncrowded thresholds (crowded – uncrowded logMAR) for active (*black solid circles*) and sham (*open circles*) a-tDCS groups. *Error bars*: ±1 SEM.

Uncrowded visual acuity, measured using the FrACT showed a significant main effect of time, *F*(2, 36) = 3.49, *P* = 0.04. Both groups exhibited improved acuity at 1 day post-training compared to baseline (0.56 ± 0.07 logMAR vs. 0.49 ± 0.06 logMAR; *P* = 0.04), but no significant differences were observed between baseline and at 1 month post-training (0.53 ± 0.06 logMAR; *P* = 0.85) or between 1 day and 1 month post-training (*P* = 0.40). No significant group effect, *F*(1, 18) = 1.08, *P* = 0.31, or time × group interaction, *F*(2, 36) = 2.59, *P* = 0.09, was observed (Fig. 4A). In contrast, crowded visual acuity showed no significant effects of time, *F*(2, 36) = 1.28, *P* = 0.29; group, *F*(1, 18) = 0.43, *P* = 0.52; or time × group interaction, *F*(2, 36) = 1.42, *P* = 0.25 (0.70 ± 0.08, 0.67 ± 0.08, and 0.66 ± 0.07 logMAR at pre-training and at 1 day and 1 month post-training, respectively) (Fig. 4B). To evaluate changes in visual crowding, the difference between crowded and uncrowded acuity was compared using the ART ANOVA due to the violation of the normality assumption at pre-training (*P* < 0.001) and at 1 month post-training assessment (*P* < 0.001) in the active and sham a-tDCS groups, respectively. No significant effect of time, ART *F*(2, 36) = 2.84, *P* = 0.07; group, ART *F*(1, 18) = 1.02, *P* = 0.33; or time × group interaction, ART *F*(2, 36) = 2.06, *P* = 0.14, was observed (0.14 ± 0.03, 0.18 ± 0.03, and 0.13 ± 0.03 logMAR at pre-training and at 1 day and 1 month post-training assessments, respectively) (Fig. 4C).

**Figure 5.**
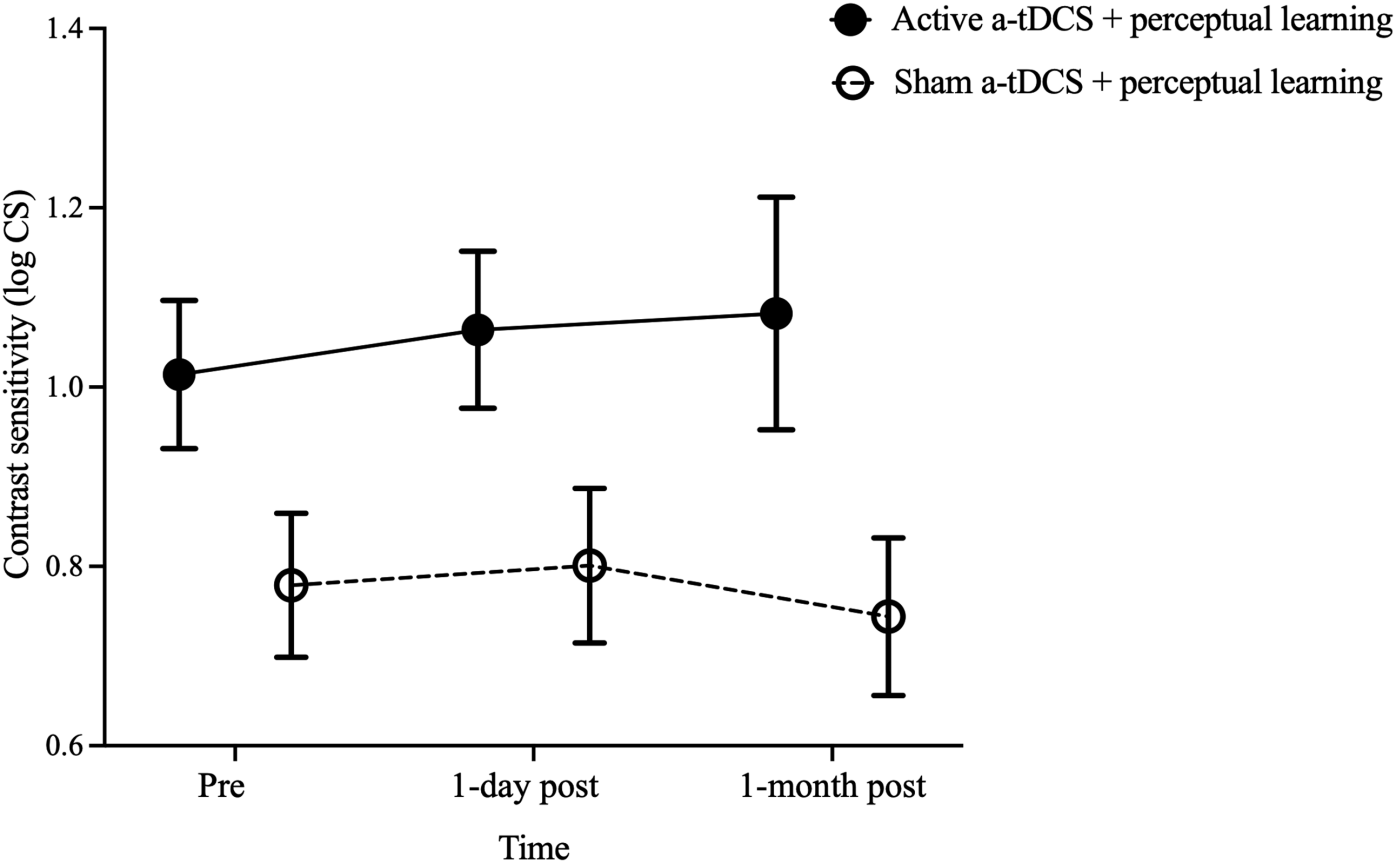
Comparison of contrast sensitivity before and after training in the active and sham a-tDCS groups. The contrast sensitivities before training (pre), and 1-day and 1 month post-training are shown. The black solid and open circles represent the mean contrast sensitivity for the groups that received active and sham a-tDCS, respectively. *Error bars*: ±1 SEM.

Contrast sensitivity measured using the FrACT revealed a significant main effect of group, *F*(1, 18) = 4.96, *P* = 0.04, with the active a-tDCS group demonstrating higher sensitivity compared to the sham group (1.05 ± 0.06 vs. 0.77 ± 0.05 log CS). No significant main effect of time, *F*(2, 36) = 0.44, *P* = 0.65, or time × group interaction, *F*(2, 36) = 0.95, *P* = 0.40, was observed (active a-tDCS, 1.01 ± 0.08 log CS vs. 1.06 ± 0.09 log CS vs. 1.08 ± 0.13 log CS; sham a-tDCS, 0.78 ± 0.08 log CS vs. 0.80 ± 0.09 log CS vs. 0.74 ± 0.09 log CS at pre-training and at 1 day and 1 month post-training assessments, respectively) (Fig. 5).

**Figure 6.**
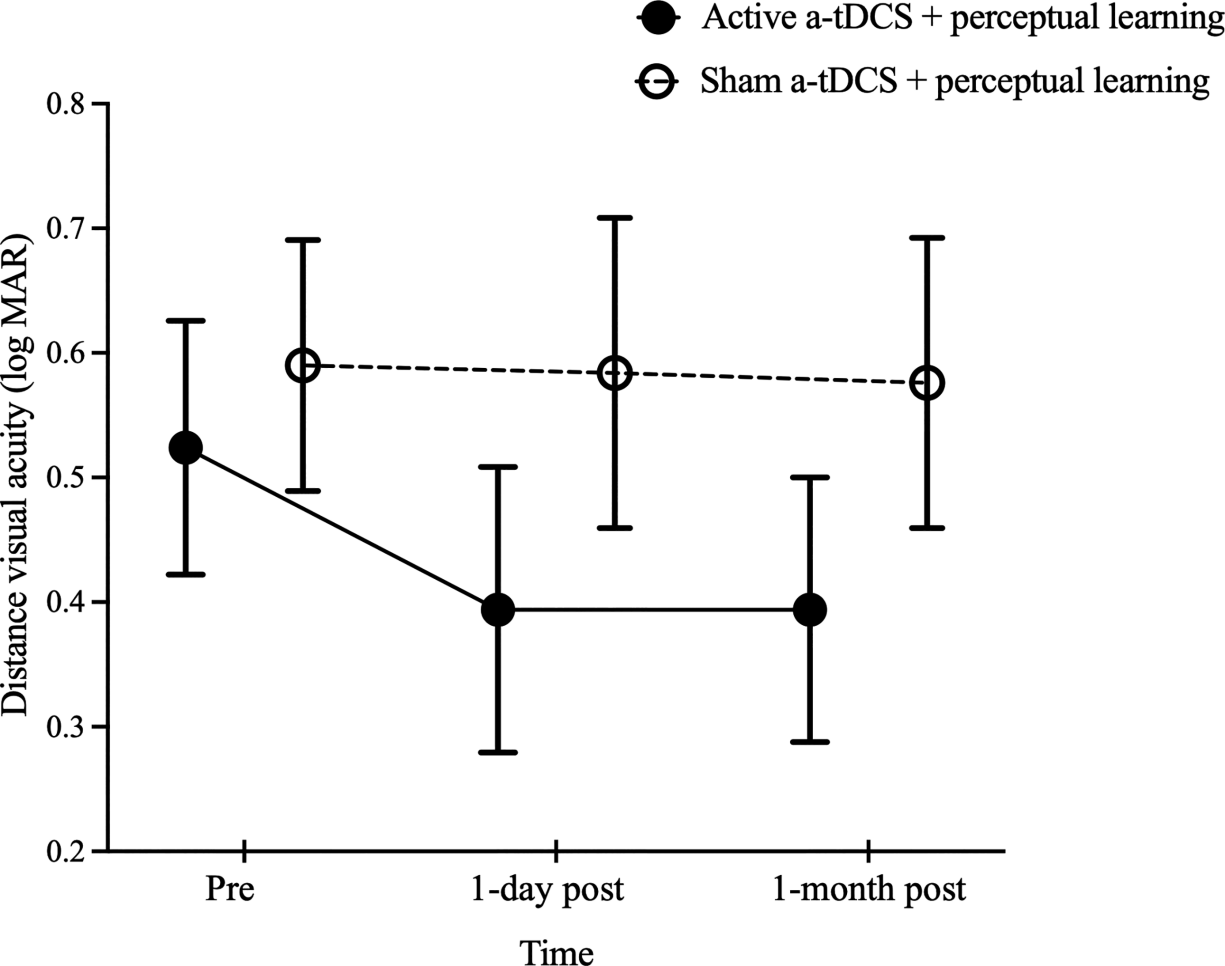
Comparison of distance visual acuity measured using the ETDRS charts before and after training in the active and sham a-tDCS groups. The visual acuities before training (pre), and at 1 day and 1 month post-training are shown. The *black solid* and *open circles* represent the mean visual acuity for the groups that received active and sham a-tDCS, respectively. *Error bars*: ±1 SEM.

Distance visual acuity measured using the ETDRS charts was compared to evaluate changes in vision over the study period. No significant group differences in visual acuity were observed, *F*(1, 18) = 0.90, *P* = 0.35. However, a significant effect of time was found, *F*(2, 36) = 3.95, *P* = 0.03, whereas the time × group interaction effect approached significance, *F*(2, 36) = 2.91, *P* = 0.07. Post hoc analyses revealed that participants in the active a-tDCS group demonstrated improved visual acuity at 1 day post-training (0.39 ± 0.11 logMAR) and at 1 month post-training (0.39 ± 0.11 logMAR) compared to baseline (0.52 ± 0.10 logMAR; *P* = 0.01). In contrast, the sham a-tDCS group showed no significant differences across time points (0.59 ± 0.10 logMAR vs. 0.58 ± 0.12 logMAR vs. 0.58 ± 0.12 logMAR; *P* > 0.99) (Fig. 6). Individual participant data for primary and secondary outcomes are provided in the Supplementary Material Figures S4 through S7.

## Discussion

This study investigated the effects of a-tDCS combined with perceptual learning on Chinese reading performance in patients with macular degeneration. The primary findings revealed that, although perceptual learning significantly improved RSVP reading speed, active a-tDCS did not enhance the training benefits compared to sham stimulation. In addition, the transfer effects of perceptual learning were limited to MNRead CPS, with ETDRS acuity improvements observed only in the active a-tDCS group. There were no generalizations to other untrained visual functions, including sentence reading speed, crowding effect, and contrast sensitivity.

Consistent with previous studies, the current results demonstrated that repetitive, task-specific training improved reading performance in individuals with macular degeneration.^19^^,^^27^^,^^28^^,^^47^ In the present cohort of Chinese readers, six sessions of RSVP training resulted in a 6% increase in log MRS. The absolute gains varied with baseline performance: Using a median split (2.35 log cpm), participants with faster baseline speeds (>2.35 log cpm; *n* = 10 [active, 7; sham, 3]) showed larger absolute improvements (mean ± SEM, 70.90 ± 29.99 cpm), whereas slower readers (≤2.35 log cpm; *n* = 10 [active, 3; sham, 7]) exhibited smaller gains (61.97 ± 18.38 cpm). These improvements, however, were comparatively smaller than the 53% increase in log speed previously reported in English readers by Chung.^19^ This difference may arise from the greater spatial complexity of logographic Chinese characters (mean stroke count = 8.69 ± 1.11 used in the current study), which introduces unique within-character crowding effects,^11^^,^^48^^,^^49^ imposing higher perceptual demands than alphabetic systems. The observed MRS improvements in both groups likely reflect a combination of adaptive neuroplasticity in the visual cortex and optimizations in oculomotor control,^50^ enabling patients to more effectively use residual peripheral vision for character recognition. Such plasticity may involve enhanced integration of parafoveal visual inputs to compensate for central scotomas.^19^^,^^51^^,^^52^ Alternatively, participants may have developed a more stable fixation with their PRL, further contributing to improved reading performance.^50^ Although these findings demonstrate the capacity for functional adaptation in individuals with macular degeneration, it is important to note that such changes can be influenced by various factors, including age.^18^ However, this was not the primary focus of the current study, and future research with larger sample sizes is warranted to investigate whether age moderates the response to perceptual learning and neuromodulation in patients with macular degeneration.

Nevertheless, in the present study, active a-tDCS applied over the primary visual cortex did not provide additional benefits, contrasting with its reported effects on alphabetic reading (Italian) in dyslexia.^53^ This discrepancy may stem from fundamental differences in visual processing demands. Chinese characters, with their high visual and cognitive complexity due to logographic structures, may be less responsive to a-tDCS. Previous research has shown that a-tDCS preferentially enhanced performance in low-complexity tasks (e.g., object recognition) over high-complexity tasks (e.g., orientation discrimination),^54^ aligning with the current results. The use of a fixed occipital electrode placement at Oz, chosen for consistency and to stimulate the general visual cortex, facilitates the comparison of a-tDCS effects between alphabetic (English) and logographic (Chinese) scripts. However, this montage (anode at Oz and cathode on cheek), previously employed in the study on a-tDCS and reading in macular degeneration,^22^ may also influence the outcomes. Participants’ PRLs were located in various regions of the peripheral visual field, corresponding to lateralized cortical representations. An individualized montage tailored to the hemisphere associated with the PRL (e.g., O2 for a left visual field PRL) could have provided more targeted stimulation to the specific cortical area processing the trained stimuli. A standardized Oz montage may have delivered a suboptimal stimulation dose to these lateralized cortical regions, reducing the potential effect of a-tDCS. Furthermore, the stimulation protocol (six sessions of 2-mA a-tDCS) may have been insufficient to induce sustained cortical excitability in patients with long-standing cortical reorganization.^18^ A previous study on dyslexia where a-tDCS improved reading efficiency involved longer stimulation periods (18 sessions across 6 weeks),^53^ suggesting that dosage and duration are critical variables. Additionally, the RSVP reading task trained within-character processing in isolation, and not crowding, which suggests that the potential benefits of a-tDCS are limited to this particular component of reading.^23^ It is noteworthy that, despite randomization with stratification for visual acuity and macular degeneration type, the active a-tDCS group exhibited possibly higher baseline performance on contrast sensitivity. However, this difference was not statistically significant and only became apparent after unmasking. Additionally, the non-significant interaction between time and group indicates that the learning curves were parallel for both groups, suggesting that the lack of additive effect of a-tDCS was unlikely to be attributed to possible baseline differences or a performance ceiling in the active a-tDCS group.

The transfer effects in this study were limited to MNRead CPS (for perceptual learning) and ETDRS visual acuity (for active a-tDCS only), with no improvements in sentence reading speed or crowding thresholds, aligning with the task-specific nature of perceptual learning.^30^^–^^32^ Training with RSVP reading, which involves isolated character recognition, possibly refines localized neural circuits,^55^ thereby enhancing MNRead CPS. However, the transient improvement in RSVP CPS at 1 day post-training, which reverted to baseline after 1 month of no training, suggests that the short-term neural adaptations for processing small characters with dynamically adjusted sizes were not sustained. This contrasts with MNRead CPS measured under fixed print sizes. Sentence reading requires coordinated eye movements and contextual cue integration, both of which are impaired in macular degeneration due to central scotomas.^4^^,^^41^^,^^56^^,^^57^ Crowding thresholds rely on resolving peripheral visual interference,^58^ which is not addressed by the single-character training of RSVP. The lack of transfer to these domains suggests that perceptual learning optimizes trained visual processes (e.g., character recognition) without engaging higher-level cognitive or oculomotor functions necessary for complex reading tasks. Future studies could explore using stimuli such as contextual sentences or adaptive crowding tasks to better integrate perceptual learning gains with real-world functional changes.

The improvements in ETDRS chart acuity (Fig. 6) for the active a-tDCS group and temporary improvements (at 1 day post-training) for uncrowded FrACT acuity (Fig. 4A) after training further reinforce the task-specific nature of perceptual learning. The improvements in ETDRS acuity within the active a-tDCS group suggest that a-tDCS may enhance cortical excitability by depolarizing neuronal membranes and modulating neurotransmitter levels.^21^ In macular degeneration, this increased excitability might temporarily enhance residual signals from the peripheral retina, leading to improved acuity. However, the lack of interaction effects in contrast sensitivity, sentence reading acuity, or crowding thresholds indicates that the effect of a-tDCS might be selective. Visual acuity measures high-contrast resolution and precise spatial summation, whereas contrast sensitivity, reading acuity, and crowding engage more distributed cortical networks.^59^^–^^61^ This dissociation suggests that a-tDCS of the occipital poles may selectively enhance spatial processing in early visual areas without affecting integrative higher-order functions. In contrast, transcranial random noise stimulation, another form of neuromodulation approach that alters signal-to-noise ratios through alternating current oscillations,^62^ has shown variable effects on contrast sensitivity and crowding in macular degeneration,^17^^,^^38^ suggesting the potential and variability of non-invasive brain stimulation paradigms. Despite the statistically significant changes, their clinical relevance remains uncertain due to test–retest variability in AMD patients.^63^ The lack of significant uncrowded FrACT interaction effects may be attributed to the limited sample size. Future studies with larger sample sizes are warranted to investigate whether a-tDCS can induce clinically meaningful improvements in visual acuity.

This study has several limitations. First, the small sample size (*n* = 20) and the predominance of AMD patients reflect recruitment challenges but maintain clinical relevance given the high prevalence of AMD. Second, all assessments and training were conducted monocularly. This approach controlled for variable binocular interactions and enabled isolation of the intervention effects, but it limited the direct generalizability of the findings to real-world binocular reading. In addition, although stimulation at the Oz site might affect both cerebral hemispheres, we did not assess outcomes in the untested eye or under binocular viewing conditions. As a result, we were unable to evaluate the potential interocular transfer of a-tDCS effects. The benefits of perceptual learning and neuromodulation may differ under binocular viewing conditions due to binocular summation and interocular suppression.^64^ Future studies should investigate whether the observed effects translate to binocular reading performance. Third, the focus on Chinese reading limits direct comparisons to alphabetic scripts, where the effect of a-tDCS remains inconsistent.^22^ Fourth, although the six-session intervention is clinically feasible, it may not capture the long-term effects of combined perceptual learning and neuromodulation. The limited transferability further highlights the need for extended training durations. Fifth, we did not characterize the precise size and location of the central scotoma for each participant, which is known to affect reading performance and crowding effects.^65^^–^^67^ Future studies would benefit from incorporating detailed perimetric mapping to assess whether scotoma characteristics predict responsiveness to perceptual learning or neuromodulation. Furthermore, the effectiveness of participant masking was not directly assessed, although the absence of group differences on the primary outcome makes a systematic bias unlikely. Future studies that evaluate participants’ perceptions of their group assignment would help validate the masking protocol. Finally, although RSVP was used in this study for its experimental controllability and precise quantification of reading speed, the observed improvements may not fully translate to gains in natural reading behaviors, which involve complex eye movements. Future studies could use sentences or paragraphs to simulate real-world reading.

In summary, perceptual learning significantly improved Chinese character reading in patients with macular degeneration. However, active a-tDCS did not provide additional enhancement effects, possibly due to the complexity of logographic characters. Future studies should explore optimized stimulation parameters, individualized approaches, or integrative training paradigms for logographic reading rehabilitation.

## Supplementary Material

Supplement 1
