# Supplementary material for: Transcranial Direct Current Stimulation Does Not Enhance Perceptual Learning of Chinese Character Reading in Adults With Macular Degeneration
Source: Invest Ophthalmol Vis Sci. 2026 Jan 8;67(1):16. doi: 10.1167/iovs.67.1.16 (PMC12805963; doi:10.1167/iovs.67.1.16)
Supplement: Supplement 1 [file iovs-67-1-16_s001.pdf]

Supplementary Material

Participants

Fifty-three individuals diagnosed with macular degeneration and fluent in reading Chinese characters were initially contacted. After screening, twenty-two participants met the eligibility criteria and consented to participate. Two participants withdrew: one due to transportation constraints and another due to relocation. The final group consisted of twenty participants (eighteen with age-related macular degeneration and two with juvenile macular degeneration) (see Supp. Figure 1).

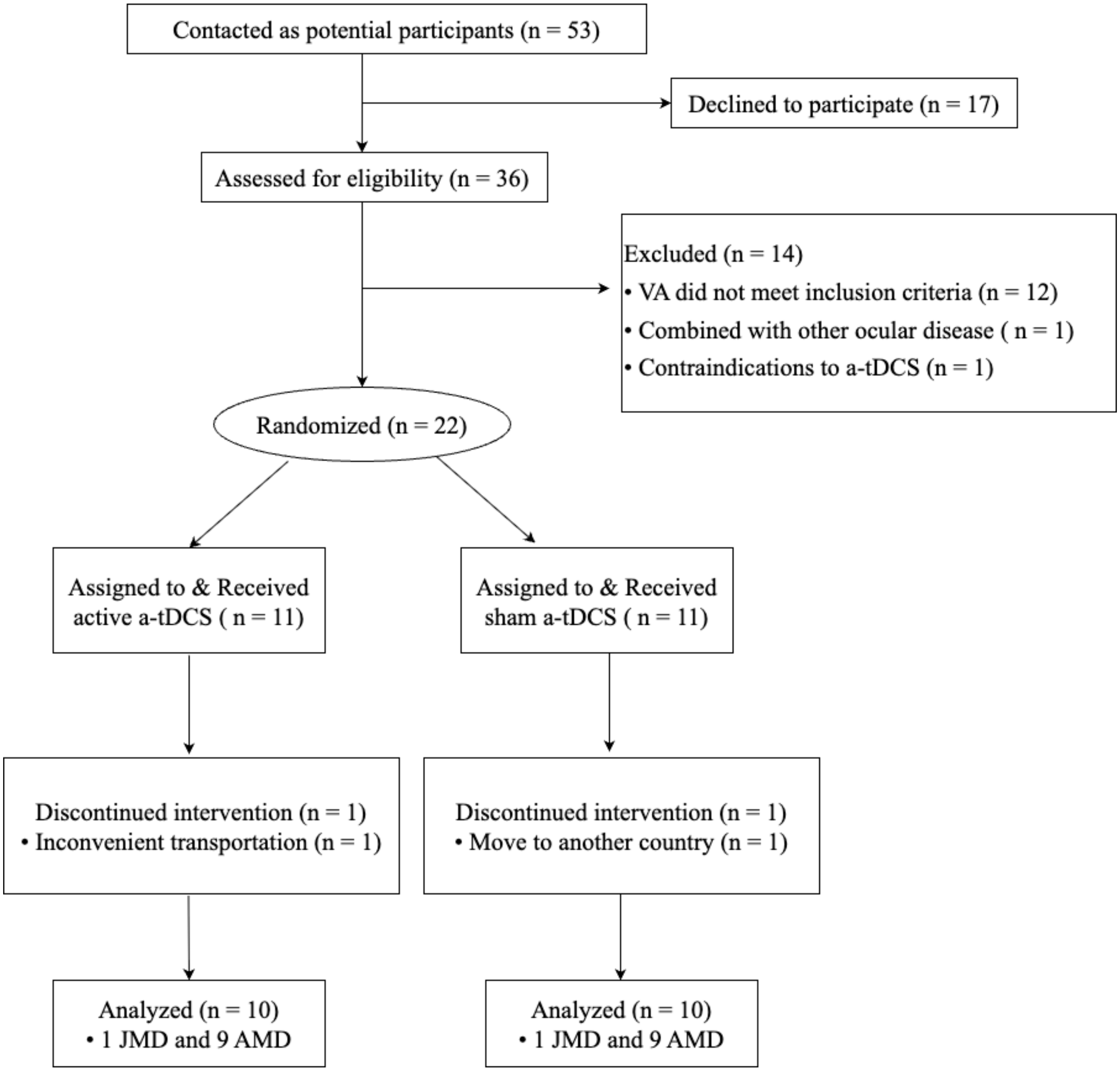

Supp. Figure 1. Recruitment and participation flow diagram of the randomized controlled trial.

Eye selection for assessments and training

The tested eye was selected based on: (1) visual acuity difference (>0.2 log MAR), prioritizing the eye with better visual acuity; (2) MNRead acuity difference (>0.2 log MAR) if visual acuity was comparable; (3) patient-reported reading preference; or (4) right eye if no preference (see Supp. Figure 2).

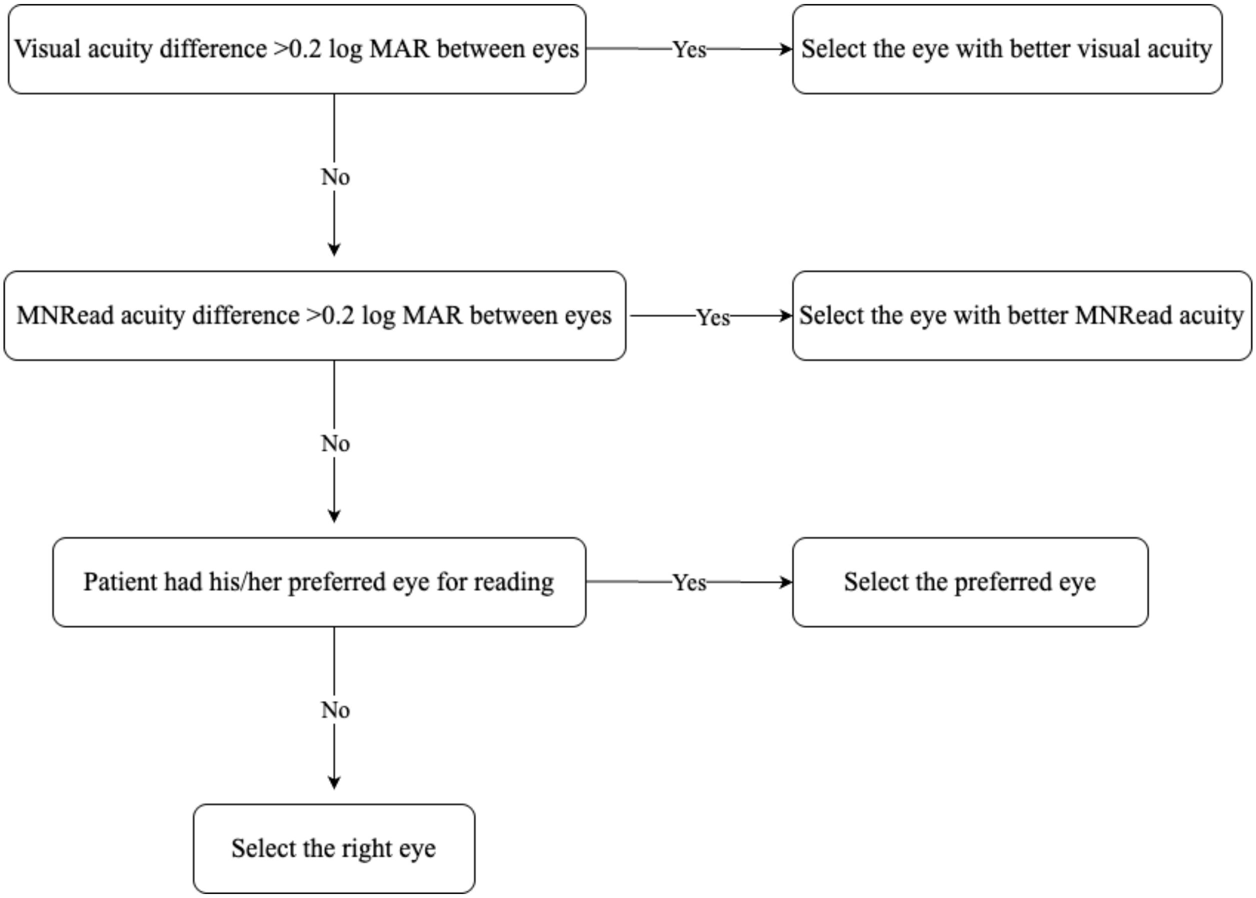

Supp. Figure 2. The process of selecting the eye to be tested for assessments and training.

RSVP curve fitting

The RSVP (rapid serial visual presentation) reading assessment was customized for each participant by adjusting the initial print size (defined as the vertical height of a square character configuration) and the testing speed (exposure duration per character). Initial parameters were determined based on MNRead maximum reading speed (MRS) and critical print size (CPS)[1]. Five exposure durations ($\frac{1}{2.25}$(0.45), $\frac{1}{1.5}$(0.67), 1, 1.5, and 2.25 times the exposure duration corresponding to MNRead MRS) were examined. Each duration was initially presented in one trial, with additional durations iteratively added at increments of 1.5 times the preceding duration until recognition accuracies fell between 20% and 80%. The print size was then adjusted in increments of ±0.16 or ±0.08 log MAR. For each new print size, exposure durations were

recalibrated by scaling the duration array of the adjacent print size by a factor of 1.3. Durations were further refined interactively as described above, adjusting until the desired range of recognition accuracy was achieved. Each participant underwent the testing with at least five different print sizes, with at least four interleaved trials per combination of print size and exposure duration, resulting in a minimum of 100 individual sentences. Recognition accuracy data were fitted to a cumulative Gaussian psychometric function to model the relationship between exposure duration and character recognition performance[2,3] (Supp. Figure 3A). The exposure durations yielding 80% recognition accuracy for each print size were plotted as log reading speed (log characters per minute, log cpm) against log print size (log MAR), and a bilinear function was applied to determine the maximum reading speed (MRS) and critical print size (CPS)[4-6] (Supp. Figure 3B).

RSVP reading during training

During the 25-minute a-tDCS block, participants performed an abbreviated RSVP protocol. Four print sizes relative to their baseline RSVP CPS were tested:

- Two smaller sizes (0.32 and 0.16 log MAR below CPS),
- One equal to CPS,
- One larger size (0.16 log MAR above CPS).

For the print size equal to CPS, four exposure durations ($\frac{1}{1.5}$(0.67), 1, 1.5, and 2.25 times the exposure duration corresponding to baseline RSVP MRS) were examined. These durations were scaled by a factor of 1.3x between adjacent print sizes and presented in an interleaved order across the first 16 trials. Additional exposure durations, adjusted by 1.5x increments, were introduced until the participant's accuracy fell between 20% and 80%. If the accuracy for the smallest print size was suboptimal, a larger print size (0.32 log MAR above baseline RSVP CPS) was used. Each final combination of print size and exposure duration was retested in three additional interleaved trials, totaling at least 64 individual sentences. Psychometric fitting was used to determine the exposure durations resulting in 80% accuracy, which were then plotted as log cpm against log MAR. A bilinear fit was applied to update the MRS and CPS, which were used to initialize the subsequent block (Supp. Figures 3C and D).

Pre-training: Baseline RSVP measurement

(A)

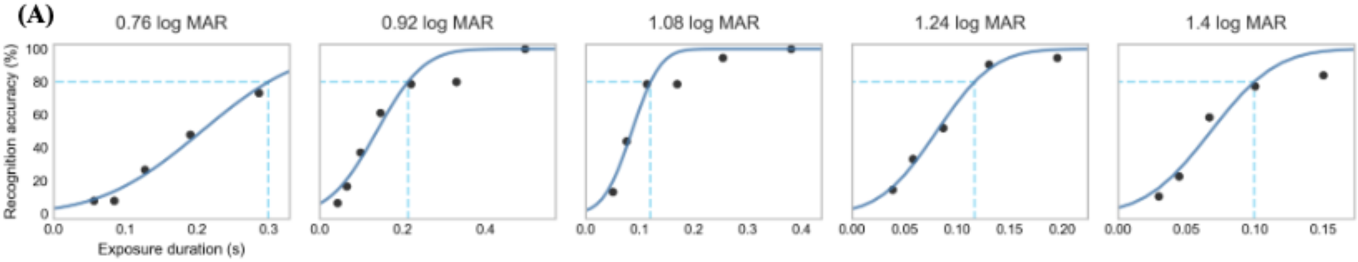

(B)

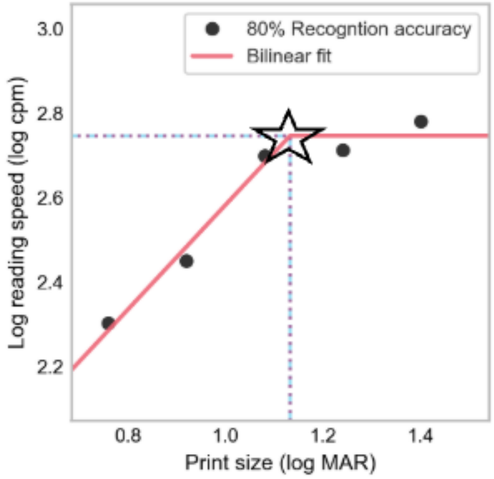

CPS and MRS were used as the initial parameters for the subsequent block of training

Training: First block of session one

(C)

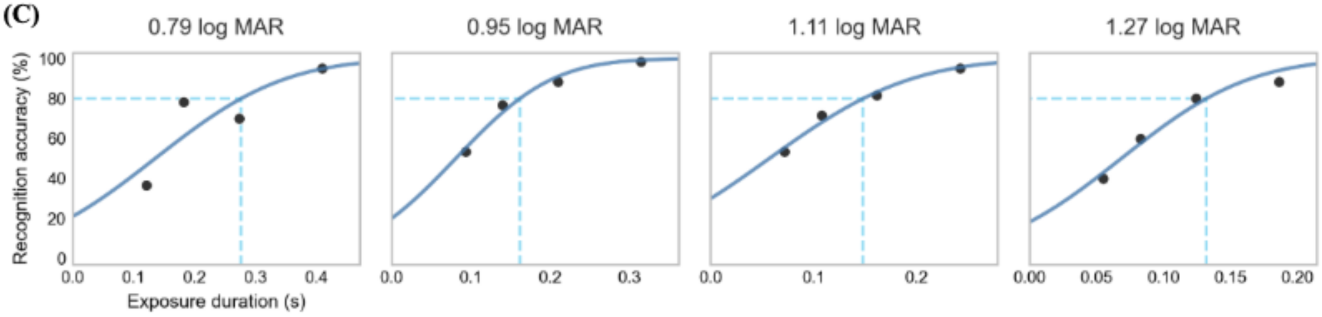

(D)

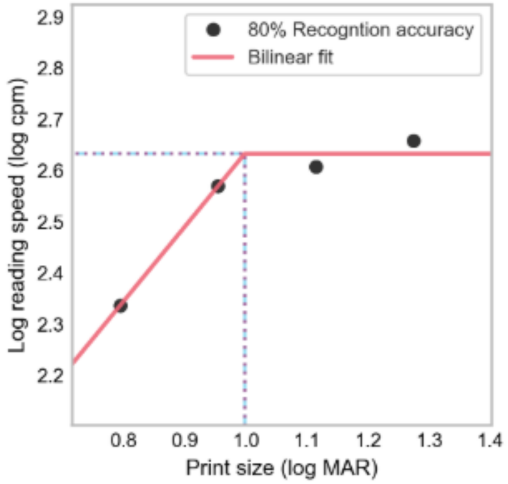

CPS and MRS were updated and used as initial parameters for the subsequent training block

Supp. Figure 3. RSVP curve fitting.

(A) Baseline cumulative Gaussian psychometric function. Recognition accuracy (black dots) at five tested print sizes (0.76, 0.92, 1.08, 1.24, and 1.40 log MAR) is plotted against exposure duration. The fitted psychometric function (blue lines) follows a cumulative Gaussian distribution. The blue dashed line indicates the exposure duration corresponding to 80% recognition accuracy.

(B) Baseline bilinear reading speed – print size relationship. Log reading speed (dark circles) is fitted to a bilinear function across tested print sizes. Dashed horizontal and vertical lines indicate the maximum reading speed (MRS; 2.75 log characters per minute) and critical print size (CPS; 1.11 log MAR), derived from the 80% recognition accuracy threshold.

(C) Training session 1 (Block 1) psychometric function. Abbreviated RSVP testing at four print sizes: one equal to baseline CPS (1.11 log MAR), two smaller sizes (0.79 and 0.95 log MAR), and one larger size (1.27 log MAR). Recognition accuracy (black dots) and the fitted cumulative Gaussian curve (blue lines) are shown. The dashed blue line marks the 80% accuracy exposure duration.

(D) Training session 1 (Block 1) bilinear fit. Exposure durations at 80% accuracy were refitted to update the reading speed - print size relationship. Resulting CPS and MRS were used as the initial print size and testing speeds for Block 2 of the training session 1.

Chinese character and sentence selection criteria

The sentences used for the RSVP training and assessment were selected from third-grade primary school textbooks to control for linguistic complexity.

Selection process:

1. The initial pool contained 2205 sentences, which were evaluated for grammatical correctness and structural validity by a Chinese teacher and clinical educator, reducing the number to 1786 sentences.
2. The remaining sentences were then read by a group of 13 normally-sighted adults, with each sentence displayed in a single line at 1.0 log MAR on a screen. Sentences with reading speeds that were statistical outliers (exceeding ±2 standard deviations (4.08) from the mean speed of ~96.29 characters per minute) were excluded, resulting in a final validated pool of 1687 sentences for use in the study.

Spatial complexity of characters:

The spatial complexity of Chinese characters, which is a key factor influencing within-character crowding, was quantified by calculating the stroke count for characters in the final pool.

- Mean stroke count: 8.69
- Standard deviation: 1.11
- Range: 1 to 28 strokes

Individual participant data plots

To supplement the group-level analyses presented in the main text, the following figures provide a detailed visualization of outcome measures for each individual participant across the study timeline.

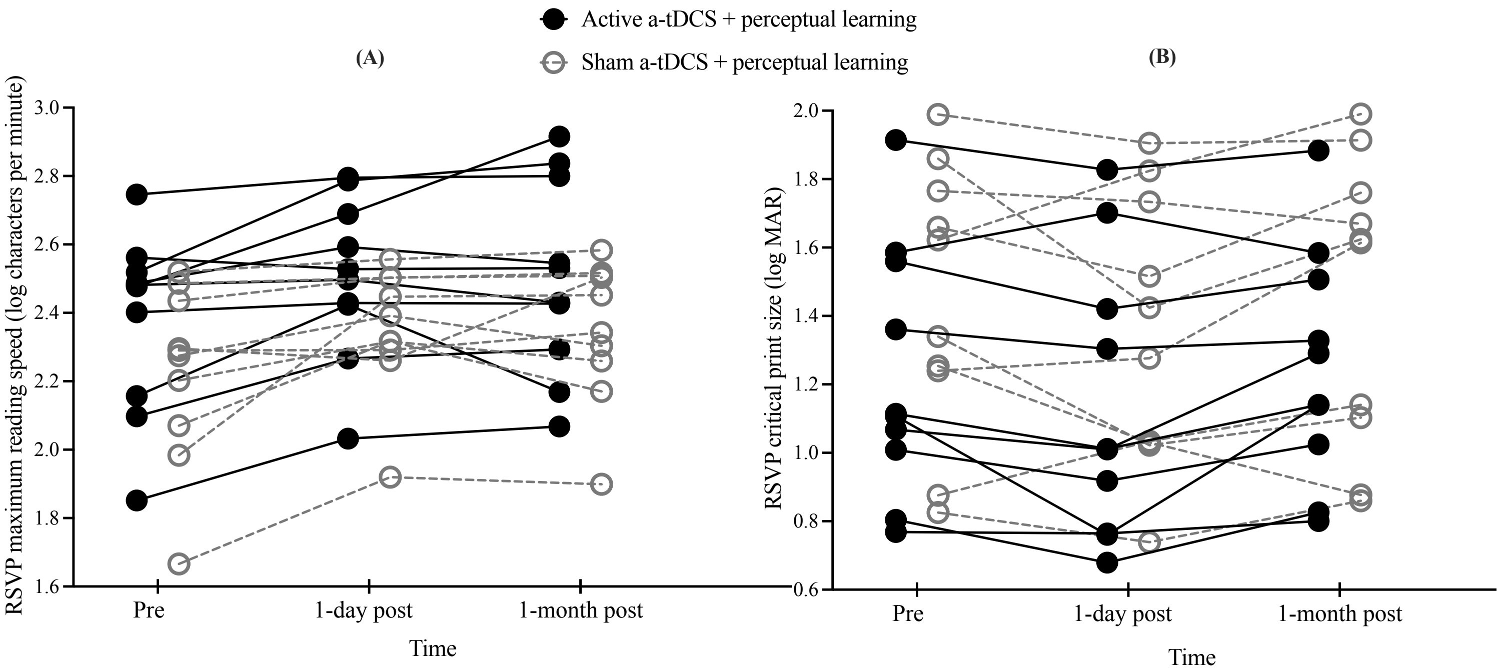

Supp. Figure 4. Individual pre- vs. post-training comparison of RSVP reading performance.

(A) RSVP maximum reading speed. Individual RSVP maximum reading speed (MRS) before and after training for the active (black solid circles) and sham (gray open circles) a-tDCS groups.

(B) RSVP critical print size. Individual RSVP critical print size (CPS) before and after training for the active (black solid circles) and sham (gray open circles) a-tDCS groups.

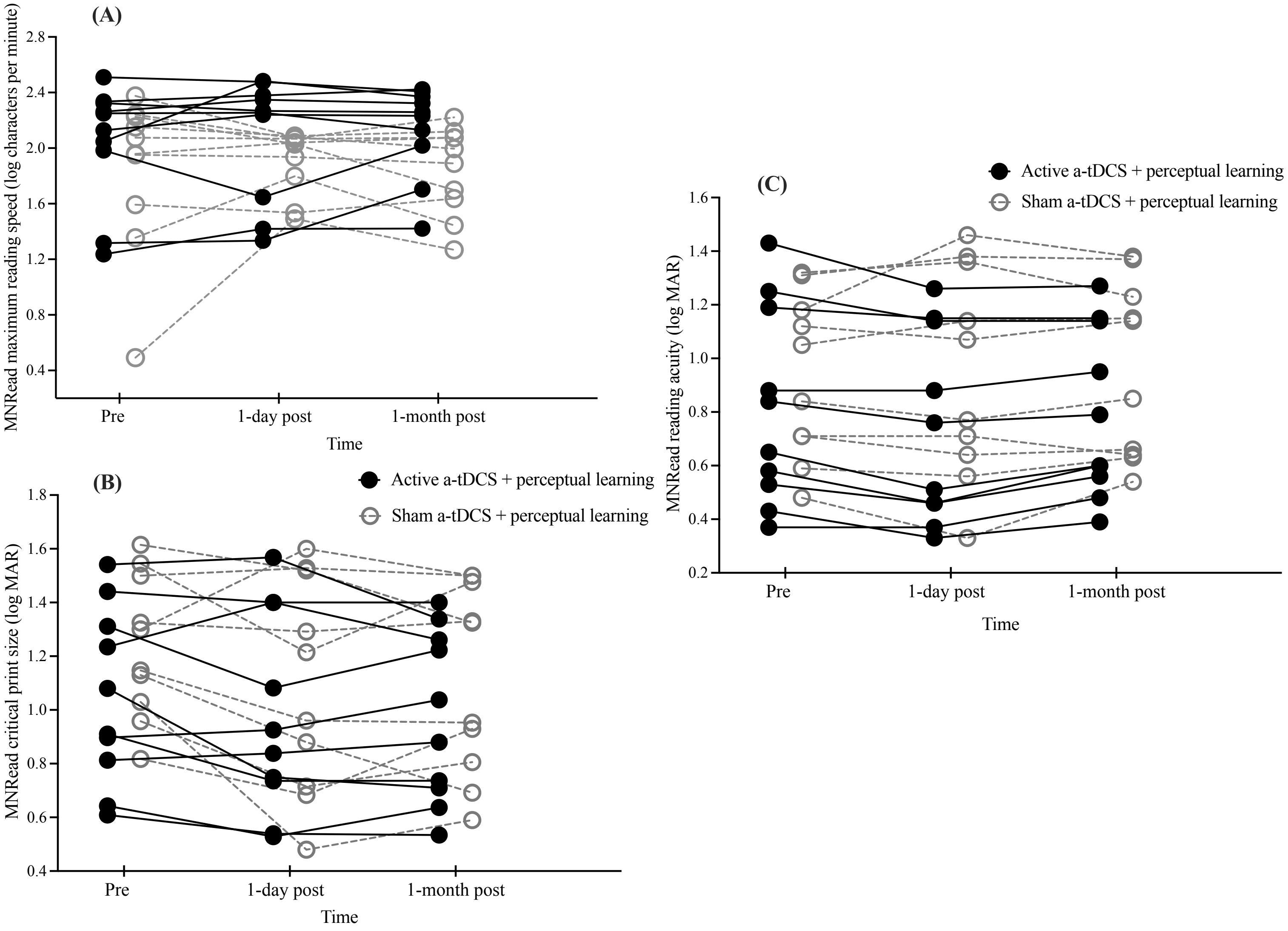

Supp. Figure 5. Individual pre- vs. post-training comparison of MNRead reading performance.

(A) MNRead maximum reading speed. Individual MNRead maximum reading speed (log MRS) before and after training for the active (black solid circles) and sham (gray open circles) a-tDCS groups.

(B) MNRead critical print size. Individual MNRead critical print size (log MAR) before and after training for the active (black solid circles) and sham (gray open circles) a-tDCS groups.

(C) MNRead reading acuity. Individual MNRead reading acuity (log MAR) before and after training for the active (black solid circles) and sham (gray open circles) a-tDCS groups.

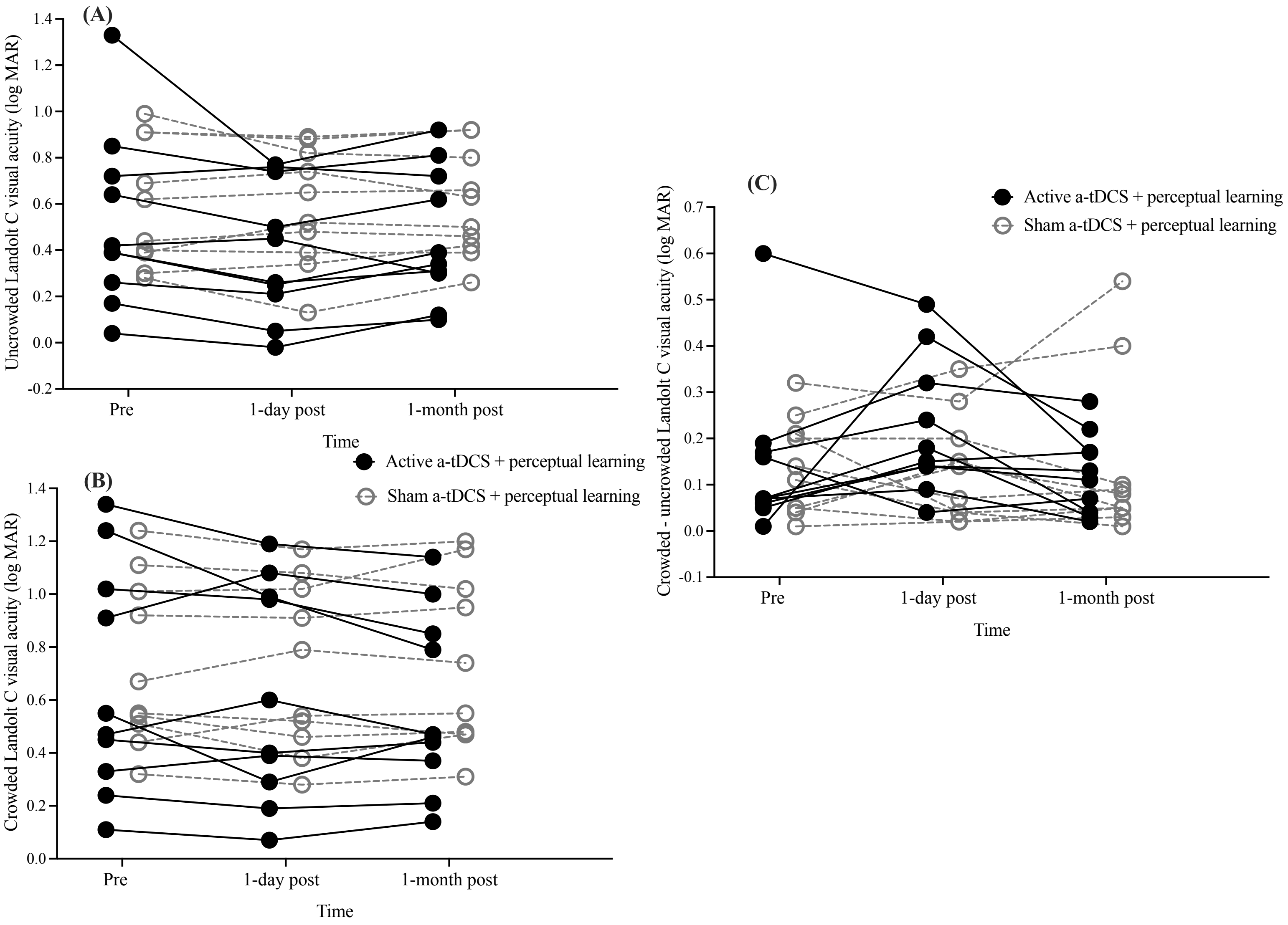

Supp. Figure 6. Individual pre- vs. post-training comparison of uncrowded acuity, crowded acuity, and crowding effect with the Freiburg Vision Test (FrACT).

(A) Uncrowded visual acuity. Individual Landolt C recognition thresholds (log MAR) before and after training for active (black solid circles) and sham (gray open circles) a-tDCS groups.

(B) Crowded visual acuity. Individual Landolt C recognition thresholds (log MAR) before and after training for active (black solid circles) and sham (gray open circles) a-tDCS groups under crowded conditions.

(C) Crowding effect. Individual difference between crowded and uncrowded thresholds (crowded – uncrowded log MAR) for active (black solid circles) and sham (gray open circles) a-tDCS groups.

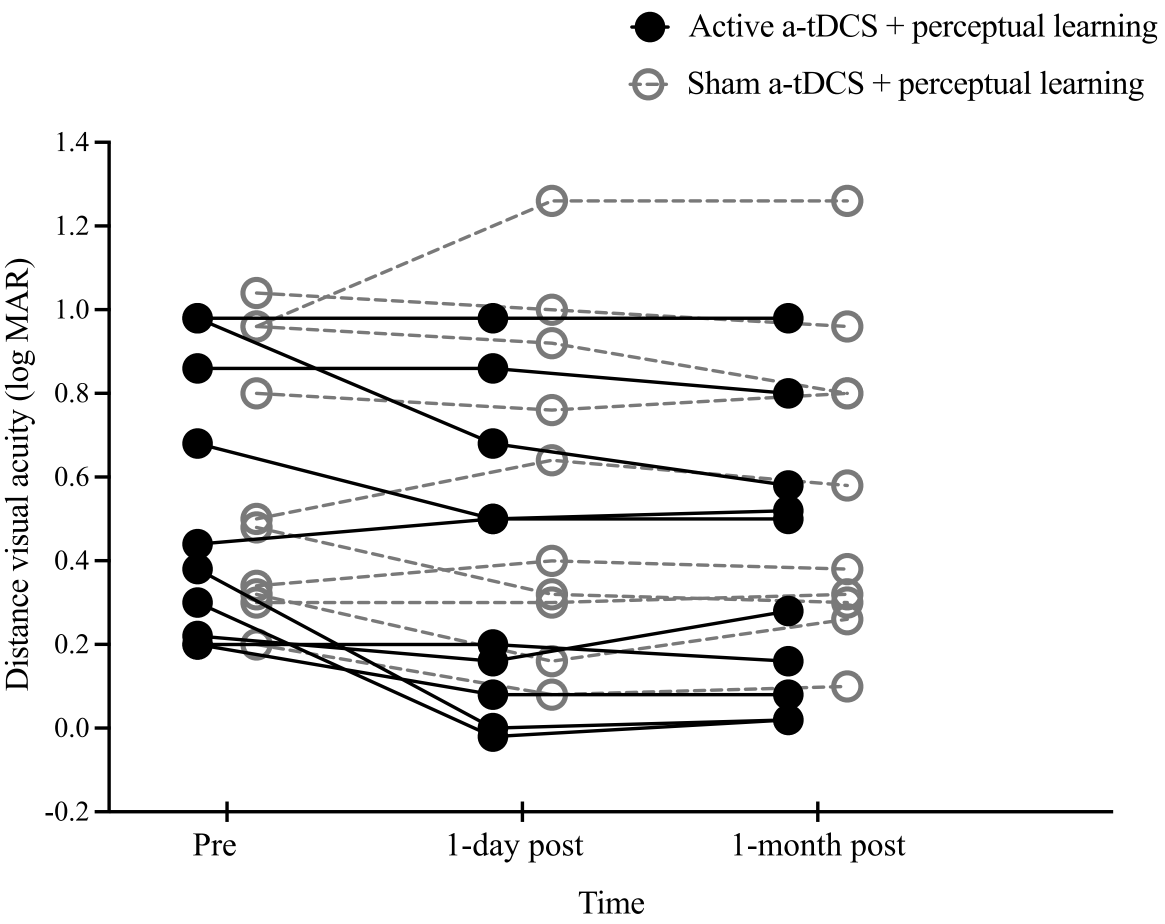

Supp. Figure 7. Individual pre- vs. post-training comparison of distance visual acuity measured using the ETDRS charts for the active (black solid circles) and sham (gray open circles) a-tDCS groups.

References

1. Cheong AMY, Legge GE, Lawrence MG, Cheung SH, Ruff MA. Relationship between slow visual processing and reading speed in people with macular degeneration. *Vis Res*. 2007;47(23):2943-2955.
2. Wichmann FA, Hill NJ. The psychometric function: I. Fitting, sampling, and goodness of fit. *Percept Psychophys*. 2001;63(8):1293-1313.
3. Wichmann FA, Hill NJ. The psychometric function: II. Bootstrap-based confidence intervals and sampling. *Percept Psychophys*. 2001;63(8):1314-1329.
4. Lyu A, Silva AE, Cheung SH, Thompson B, Abel L, Cheong AMY. Effects of visual span on Chinese reading performance in normal peripheral vision. *Vis Res*. 2022;201:108119.
5. Chung STL. Improving reading speed for people with central vision loss through perceptual learning. *Invest Ophthalmol Vis Sci*. 2011;52(2):1164-1170.
6. Chung STL. Training to improve temporal processing of letters benefits reading speed for people with central vision loss. *J Vis*. 2021;21(1):14-14.
